# Behaviour, Attitudes and Knowledge of Healthcare Workers on Breastfeeding

**DOI:** 10.3390/children9081173

**Published:** 2022-08-05

**Authors:** Marija Čatipović, Zrinka Puharić, Drita Puharić, Paula Čatipović, Josip Grgurić

**Affiliations:** 1 Department of Nursing, Bjelovar University of Applied Sciences, TrgE.Kvaternika 4, 43000 Bjelovar, Croatia; 2 Specialist Gynecological Practice Marija Divić, 21000 Split, Croatia; 3 Pediatric Office MarijaČatpović, 43000 Bjelovar, Croatia; 4 UNICEF Office for Croatia, 10000 Zagreb, Croatia

**Keywords:** breastfeeding behavior, attitudes, knowledge, healthcare professionals

## Abstract

The aim of this paper is to determine the current state of behavior, attitudes, and knowledge of health professionals about breastfeeding in Croatia. Data were collected via a breastfeeding behavior, attitudes, and knowledge questionnaire, which has already been validated and used in Croatia. The secondary aim is to identify differences in outcomes of respondents by occupation (nurses versus others health professionals). In the study, 374 health professionals participated (37 males and 337 females). Respondents completed the questionnaire online. Respondents were rarely involved in breastfeeding education. On the behavior scale, the worst answer was given to the question of advising mothers on breastfeeding after 24 months. On the attitude scale, the worst result was achieved in terms of public breastfeeding and the support of the child’s father for the breastfeeding mother. Respondents demonstrated the worst knowledge of The International Code of Marketing of Breast-milk Substitutes and the use of medications while breastfeeding. There was no statistically significant difference between the results of respondents in relation to the occupation of the respondents. In the preparation of future breastfeeding education for health professionals in Croatia, particular attention should be given to these issues.

## 1. Introduction

According to the 2018 UNICEF Status of Children Report, improving breastfeeding rates worldwide could save hundreds of thousands of children’s lives each year [1]. Children who eat artificial food have an increased risk of hospitalization due to the development of lower respiratory tract diseases in the first year of life, acute ear infections, diarrhea and vomiting, asthma (with positive familial anamnesis), type 2 diabetes, sudden infant death syndrome, eczema (atopic dermatitis), asthma (with no positive familial anamnesis), children obesity, acute lymphocytic leukemia, and acute myeloid leukemia, while in premature infants, there is also necrotizing enterocolitis [2]. Breastfeeding has been shown to protect against postpartum depression [3], breast cancer [4], ovarian cancer [5], endometrium [6], cardiovascular diseases [7], arterial hypertension [8], and type 2 diabetes [9]. More than 800,000 lives of children under 5 years old can be saved each year by improving breastfeeding practices globally [10]. What should also be added is the twenty thousand lives of mothers who are saved from breast cancer [11]. In addition, there are huge economic losses due to non-breastfeeding [12,13], and breastfeeding is definitely environmentally acceptable [14,15]. The WHO and UNICEF recommend the first breastfeeding immediately after birth, exclusive breastfeeding for the first six months of the child’s life and breastfeeding with adequate nutrition for up to two years or longer [16].

Despite all this, data on breastfeeding aren’t satisfactory either globally or in Croatia. According to UNICEF data for the whole world, only 48 percent of infants are breastfed within the first hour of life, and 44% of mothers exclusively breastfeed for 6 months [17]. According to the Croatian Health Statistics Yearbook for 2019, in the group of children aged 90 to 179 days, 33.5% of reports lacked data on nutrition, 57.2% of infants were breastfed exclusively, and 18.4% of infants were breastfed with supplementary feeding and/or substitutes [18].

There are many factors that affect the success of breastfeeding [19,20]. More and more attention is paid to the influence of health professionals on parents’ decision to breastfeed [21]. The Surgeon General of the United States stated that among the biggest obstacles to successful breastfeeding is inadequate education and training of health professionals. The Surgeon General recommended continuous training and education of health professionals but also periodic licensing and certification of knowledge and skills of health professionals in order to ensure satisfactory quality of breastfeeding support [2]. The problem that arises in assessing the procedures, attitudes, and knowledge of health professionals about breastfeeding is the application of an objective measuring instrument that will allow comparison of results and evaluation of the effectiveness of educational interventions. 

Several questionnaires examining the attitudes and knowledge of breastfeeding health professionals are currently available online, such as the Health Professional Breastfeeding KAP questionnaire [22], a questionnaire used in a national survey of infant feeding practices at hospitals and birth centers in the United States [23], Perceptions of Workplace Breastfeeding Experience Questionnaire (PWBE-Q) [24], etc. However, the use of other authors’ questionnaires is burdened by the issue of permission to use them, and if it is a questionnaire from another language area, there is a problem of official translation and adjustment of the questions due to cultural, economic, social, religious, and other specifics of the country in which the questionnaire will be used [25,26]. In this study, the Breastfeeding Behavior Attitudes and Knowledge Questionnaire (BBAKQ prof.) was used because it has been validated and used in Croatia since 2020.

The primary aim of this paper is to determine the current state of behavior, attitudes, and knowledge of health professionals in Croatia. According to data from a survey of the situation in some Croatian maternity hospitals, the situation is not satisfactory. A 20 h course on breastfeeding held once a year for two years did not result in a significant change in the rate of breastfeeding at discharge from the maternity hospital, 3, 6, and 12 months after delivery [27]. The secondary aim is to identify differences in respondent outcomes by occupation (nurses versus others health professionals). The authors expect unsatisfactory knowledge, attitudes, and behavior of health professionals about breastfeeding, regardless of their occupation. This study is necessary because, to improve the rate of exclusive and total breastfeeding, it is necessary to improve the knowledge, attitudes, and behavior of health professionals about breastfeeding.

## 2. Materials and Methods

### 2.1. Study Design

A cross-sectional, quantitative study of the behavior, attitudes, and knowledge on breastfeeding by health professionals in the Republic of Croatia was conducted. Research materials were collected using a validated online questionnaire on the behavior, attitudes, and knowledge of health professionals on breastfeeding.

### 2.2. Measuring Instrument

The Breastfeeding Behavior Attitudes Knowledge Questionnaire consists of 9 questions about behavior, 19 questions about attitudes, and 20 questions about knowledge. Possible responses on the behavioral scale were graded on a Likert-type scale from 1 to 5 and defined the frequency of the examined behavior (1—never; 2—very rare, only a few times; 3—more than a few times, but not often; 4—often; 5—regularly). On the scale of attitudes, respondents evaluated claims on a Likert-type scale from 1 to 5 (1—strongly disagree; 2—mostly disagree; 3—neither agree nor disagree; 4—mostly agree; 5—totally agree). On the scale of knowledge, the possible answers were true and false. The survey also collected data on gender, age, and occupation. The validation process of the Breastfeeding Behavior Attitudes Knowledge Questionnaire was the subject of several diploma theses at Bjelovar University (internal coherence, exploratory, and confirmatory analysis) [28,29,30]. Considering that there is a questionnaire of behavior, attitudes, and knowledge of parents about breastfeeding (BBAKQ), the questionnaire for health professionals is called BBAKQ prof. The questionnaire is available as Supplementary Materials.

### 2.3. Data Collection Process

The questionnaire was posted on the websites of the Josip Juraj Strosmayer University in Osijek and Bjelovar University. Healthcare professionals received information about the study and the links to access the questionnaire in different ways: from the association “For a healthy and happy childhood”, direct contacts from colleagues who actively participate in breastfeeding promotion activities, through the Croatian Breastfeeding Support Association, with the assistance of lecturers from Josip Juraj Strossmayer University Osijek and Bjelovar University, and through UNICEF’s “Specialist Pediatric Clinics-Friends of Breastfeeding” [31]. The questionnaire could be completed in the period from 15 December 2020 to 15 March 2021. During this time, 374 medical staff properly completed the questionnaire. The authors intentionally did not in any way influence the response of respondents according to gender or occupation. 

### 2.4. Transformation of the Results

The scoring of the BBAKQ questionnaire was carried out in such a way that each answer was evaluated in accordance with the instructions for examiners, which are an integral part of the questionnaire. On the scales of behavior and attitudes, the best grade, grade five, scores the behavior or attitude of the respondents that is fully in line with the recommendations of the profession, and the answer completely contrary to the guidelines of the profession was scored the worst grade, one. On the knowledge scale, the correct answer is scored with one point, the incorrect answer with zero points. Scoring is performed by inverse evaluation for questions that contain negative statements.

### 2.5. Statistical Procedures

At the beginning of the study, the sample size was calculated with an online sample size calculator (https://www.calculator.net/sample-size-calculator.html) (accessed on 1 December 2020). The confidence level was 95%, the population size was 10,000 (the number of healthcare professionals working directly with newborns, infants and young children), we used the worstcase percentage (50%). The program calculated that a sample size of 370 was required. Respondent scores for the questionnaire items, age, gender, and employment data are presented using descriptive statistical methods. Differences in respondent results by occupation (nurse/other occupations) were analyzed by the Mann–Whitney U test. A significance level of 0.05 was assigned. Due to the relatively small sample size, a decision was made to post the results to one decimal place. SPSS Statistics V22.0 was used in the analysis.

## 3. Results

In the study, 374 health professionals participated (37 males and 337 females). The average age of the respondents was 37.8 years (SD 10.5). The youngest participant in the study was 20 years old, and the oldest was 76 years old. Most respondents were from Zabok (23.8%), Osijek (21.18%), Zagreb (15.7%), Slavonski Brod (8.4%), and other respondents were from various Croatian cities. The study involved 303 nurses (204 nurses/technicians of general bachelor’s degree in nursing, 99 nurses/technicians of general specialization of secondary education), 44 midwives (28 secondary education and 16 bachelors), 18 doctors (11 gynecologists, 5 pediatricians, 2 doctors of other specializations), 5 physiotherapists (1 secondary education and 4 bachelors), and pharmaceutical technicians (2), and members of other health professions (2). Out of the total number of respondents, 20.1% worked in the patronage service, 8.8% in the maternity hospital, 7.2% in the pediatric department, 2.2% in family medicine, 0.8% in the gynecological office, 0.5% in pediatric clinic, and the rest worked at other health care positions. Out of the total number of respondents, 4.8% were physicians.

Table 1 shows the respondents’ answers to the behavior scale of Breastfeeding Behavior Attitudes and Knowledge Questionnaire (BBAKQ prof.)

Respondents were rarely involved in breastfeeding education. Healthcare professionals too rarely advised mothers on breastfeeding after 24 months in accordance with UNICEF recommendations.

In Table 2 are presented the respondents’ answers to the attitudes scale of Breastfeeding Behavior Attitudes and Knowledge Questionnaire (BBAKQ prof.)

On the attitude scale, health workers achieved the worst results regarding public breastfeeding and support from the child’s father to the breastfeeding mother.

Table 3 shows the respondents’ answers to the knowledge scale of Breastfeeding Behavior Attitudes and Knowledge Questionnaire (BBAKQ prof.)

Respondents demonstrated the worst knowledge of The International Code of Marketing of Breastmilk Substitutes and the use of medications while breastfeeding.

In Table 4 are presented the respondents’ results on the Breastfeeding Behavior Attitudes and Knowledge Questionnaire (BBAKQ prof.) by respondents’ occupation.

There was no statistically significant difference between the results of respondents on individual scales of the questionnaire or the overall results of the questionnaire in relation to the occupation of the respondents (nurse/other occupations in healthcare).

## 4. Discussion

A review of the scale of respondents’ answers to the topic of behavior shows that very few respondents participate in breastfeeding education. The UNICEF/WHO Baby Friendly Hospital initiative (BFHI) recommends that all health personnel should be trained to implement best practice breastfeeding policies [32,33]. How can healthcare professionals be motivated to participate in breastfeeding education programs? In our research, we had a good response from women and nurses. Men and members of other occupations in healthcare showed a weak response to participation in the research. The disproportionality between women and men and nurses and other occupations is also found in other research on the behavior, knowledge, and attitudes of healthcare professionals about breastfeeding [34,35,36,37]. The topic of breastfeeding is often neglected and underestimated in the education of healthcare professionals [38,39]. Some colleagues mistakenly believe that there is no need to learn about breastfeeding or that there is nothing to learn because breastfeeding a purely instinctive and biological act [40]. When a healthcare professional still decides to provide breastfeeding education, he is faced with the new problem, the question of the effectiveness of individual breastfeeding educational programs [41,42].

The results of this research indicate that health professionals have a problem with the practical implementation of WHO and UNICEF guidelines on prolonged breastfeeding [43]. Since the guidelines are very clear, we are sure that healthcare professionals can understand them. An obstacle to the implementation of guidelines in practice can be a conflict of knowledge and personal attitudes. The attitudes towards breastfeeding are rooted in personal and vicarious experiences [44]. Negative attitudes can be a significant obstacle to the acquisition and application of new knowledge and skills. If health professionals themselves are not sure about the recommendations they give to parents, how convincing can they be in practice? Controversial messages about exclusive breastfeeding have been identified as a significant barrier to successful breastfeeding [45]. Mothers do not expect only theoretical knowledge from healthcare professionals. They expect expert advice, guidance, support, and encouragement [46].

By upholding traditional beliefs about gender roles, women’s autonomy and right to decide where to breastfeed is undermined. The ethical obligation to feed a hungry child must not be limited to mothers: everyone is ethically responsible for feeding children, everyone is obliged to contribute to improving social and economic support for mothers who want to breastfeed [47]. That especially goes for health professionals. According to Melanie Klein, the child’s ego is capable of experiencing anxiety from the moment of birth. The close relationship that the child had with the mother during intrauterine life replace new external relationship with the mother. The newborn expects from its mother food, love, and understanding. The immature child’s ego experiences the relation to the breast as a relation to good and bad breast. The bad breast not only fails to provide food and gratification but also threatens with destruction [48]. The newborn perceives hunger as an immediate threat to the destruction of himself and the object (breast) with which he forms a whole. Partially under the impact of the frustration and anxiety in the breast relationship, the child’s desires and fantasies extend to the whole of his mother’s body [49]. The early relationship between the child and the mother is the fundamental human relationship on which the child will build his relationships with other people in adulthood [50].

Health professionals achieved as a low percentage of positive responses regarding breastfeeding in public [51]. Newborns and young infants have the illusion that their mother’s breasts are somehow a part of them [52]. The child does not understand that the mother must not breastfeed him because he is in a public place. In these circumstances, both the child and the mother suffer, and health professionals should not support circumstances that result in the suffering of the child or the mother. Opposition to breastfeeding in public is associated with hostile attitudes towards women who are seen as violating traditional gender roles [53]. 

While breastfeeding her child, the mother will need the help of the child’s father. The UN experts have expressed a clear view on the importance of the fathers’ support in breastfeeding [54]. The personal experience health workers with breastfeeding (themselves or their partner) increased breastfeeding knowledge, attitudes, and effectiveness [55]. Health workers without practical experience in the field of breastfeeding often perceive breastfeeding as an easy and pleasant process. How painful is the child’s grip on the nipple with the gums? They do not recognize the mother’s fear after giving birth that her child will not gain enough body weight if exclusively breastfed. Without personal experience, it is difficult to imagine the psychological state in which a mother is brought by the demands of her loved ones to give her exclusively breastfed child tea or water on hot days. How does a mother feel when she objects to the child’s father’s request to soothe her with a dummy pacifier? The help that a healthcare professional provides to a nursing mother by encouraging her and positively influencing her husband and other relatives is underestimated. Breastfeeding is much more than feeding [14]. Healthcare professionals should understand breastfeeding as a biopsychosocial, dynamic, and relational process and not as an exclusively instinctive biological act [40]. Women expect health professionals to promote the positive breastfeeding experience and provide them with sensitive and individualized breastfeeding support [21].

The adoption of legislation implemented, monitored and followed by the International Code fully contributes to increasing the breastfeeding rate [56]. Many healthcare professionals do not know about the existence of the Code, which makes them susceptible to direct and indirect marketing [57]. It is also a fact that compliance with the Code is not legally mandatory in all countries. The 2020 Report highlights continued legal shortcomings in many countries [58].

The knowledge of health professionals on the use of medication during breastfeeding has been lacking [59]. When the child’s mother receives medication, some health professionals suggest stopping breastfeeding, rather than carefully studying the relationship between the medication and breastfeeding. Research conducted by other authors highlights the lack of health professionals’ knowledge about the benefits of breastfeeding for a child [37]. Lack of cognitive and emotional understanding of the benefits of breastfeeding for mother and child is the main reason why some health professionals easily give up breastfeeding support.

Female respondents had better results than male respondents on the scale of behavior, attitudes, and overall results on the questionnaire. The small number of male subjects who agreed to participate in the study precludes a reliable statistical analysis of the significance of gender-based differences in outcomes. However, the gender gap in knowledge and attitudes about breastfeeding is confirmed by the works of other authors [60,61]. The differences derive from the personal experience of a woman [62]. The first menstrual period forces the girl to think about pregnancy and childbirth. Attitudes toward breastfeeding are formed in the early years of adolescence [63,64]. Many children already in their teens think of selecting the best food for their child when they become parents [65]. That is why schools are ideal places to educate kids on breastfeeding [66]. Unlike girls, boys’ reflection on pregnancy and childbirth does not involve biological participation but focuses on supporting and assisting women. These differences should be taken into account when designing motivational and educational programs on breastfeeding.

Compared to other healthcare employees, nurses did not achieve better results on individual scales or overall results of the questionnaire. In other studies, the results are not uniform. Some studies claim that people with their bachelor’s in nursing have better knowledge about breastfeeding [34], while others claim better practical results when performed by physicians [67]. In our study, the group compared to nurses’ findings consisted primarily of midwives and physicians. When we look at it that way, the results of the nurses are great. It seems reasonable to conclude that the quality of targeted workplace education is essential. This study reinforces the idea of continuous work with health professionals from student days onwards through their practice, using well-structured breastfeeding education programs, emphasizing the importance of practical work and practical exercises [40]. Mistakes in the skills, attitudes, and knowledge of health professionals about breastfeeding have severe consequences for healthcare users, and these mistakes will not be corrected spontaneously, but with well-designed necessary educational interventions [42].

The weakness of the study is in the relatively small sample of respondents from a limited geographical, cultural, and linguistic area. 

## 5. Conclusions

Healthcare professionals do not have a satisfactory level of knowledge, positive attitudes, and breastfeeding support skills, regardless of their occupation. To improve the rates of exclusive and total breastfeeding, it is necessary to provide quality education. It is important that the education is not only focused on knowledge but also on the attitudes and skills of health professionals, which can only be achieved through personal experience of practical work with breastfeeding mothers, their children, and partners. 

## Figures and Tables

**Table 1:** Overview of the respondents’ answers to the behavior scale BBAKQ prof questionnaire (N = 374).

	Percentage of Responses					
Behavior Scale BBAKQ Prof Questionnaire	Never	Very Rare, Only a Few Times	More than a Few Times, but Not Often	Often	Regularly	Total
How often do you advise mothers to continue breastfeeding after 6 months with supplementation?	9.9	10.7	13.6	24.9	40.9	100.0
How often do you participate in breastfeeding education?	42.8	20.3	14.2	12.3	10.4	100.0
How often do you advise mothers to continue breastfeeding after 24 months of the child’s life, if both mother and child want it?	22.2	12.0	15.8	19.3	30.8	100.0
How often do you advise mothers to exclusively breastfeed for 6 months?	20.1	10.4	11.5	18.5	39.6	100.0
How often do you advise giving tea to a breastfed baby in the first 6 months of life?	56.2	24.6	11.5	5.4	2.4	100.0
How often do you advise breastfeeding mothers to stop breastfeeding when introducing any drugs (e.g., penicillin, cephalosporins, paracetamol…)	46.5	21.1	12.8	10.4	9.1	100.0
How often do you automatically recommend that a mother stop breastfeeding because shi is feverish?	53.7	23.5	8.8	6.4	7.5	100.0
How frequently do you advise mothers to stop breastfeeding if ragada occurs on one breast?	66.8	17.1	9.6	2.4	4.0	100.0
How often do you advise giving a pacifier to a breastfed baby in the first 6 months of life?	46.2	27.8	16.2	6.8	3.0	100.0

**Table 2:** Overview of the respondents’ answers to the attitudes scale BBAKQ prof questionnaire (N = 374).

	Percentage of Responses					
Attitudes Scale BBAKQ Prof Questionnaire	Strongly Disagree	Mostly Disagree	Neither Agre nor Disagree	Mostly Agree	Totally Agree	Total
Breastfeeding should not be viewed solely as a process of satisfying hunger, but as a mechanism to reduce the stress and discomfort of the child and mother.	7.0	4.3	9.9	17.4	61.5	100.0
Mothers who breastfeed for more than a year are exhausted and susceptible to disease.	67.1	13.9	10.4	3.2	5.4	100.0
Breastfeeding is only needed in poor countries where children die of starvat ion.	87.2	7.2	1.6	0.8	3.2	100.0
It is in the best interest of the child and the father that each father spends as much time as possible with the child and if he is employed use his right to two months of parental leave.	5.1	4.0	9.4	22.2	59.4	100.0
Breastfeeding negatively affects the mother’s ability to work.	72.5	16.0	6.2	1.9	3.5	100.0
It is right and natural for a mother to breastfeed a hungry child in a public place.	7.5	2.9	8.0	19.5	62.0	100.0
I don’t understand men who help women with breastfeeding, such behavior is completely “non-male” to me.	78.3	8.8	6.7	2.4	3.7	100.0
Breastfeeding is a mother’s way of providing comfort, love, security and communication with the child.	5.4	2.9	6.7	12.8	72.2	100.0
Breastfeeding disrupts a partner’s intimacy.	70.6	13.9	11.5	1.9	2.1	100.0
The obligation of society is to provide every future parent with information about the benefits of breastfeeding.	4.8	3.5	6.4	10.4	74.9	100.0
In an age of respect for differences and personality rights, a mother’s right to breastfeed a hungry child in public should be respected.	5.9	4.8	8.3	16.0	65.0	100.0
No matter what medicine says about the justification of breastfeeding after a year, I think that such prolongation of breastfeeding has harmful consequences not only for individuals but also for society as a whole.	66.8	11.5	13.6	3.2	4.8	100.0
If I had any power in the state, I would protect the right of the child to be breastfed when he is hungry, because it is the fundamental right of every human being not to suffer hunger.	8.0	4.0	9.4	11.2	67.4	100.0
At the time the mother is breastfeeding the role of the father is not significant.	75.9	10.7	5.9	2.9	4.6	100.0
A man disrupts his male role in the family when he helps a woman in the house and around breastfeeding.	84.8	8.0	3.2	1.1	2.9	100.0
I don’t understand people who admire naked women’s breasts and are disgusted by any idea of breastfeeding in a public place.	7.8	5.1	12.3	12.6	62.3	100.0
Mothers who breastfeed for more than a year are at risk of spoiling their children.	71.1	12.6	9.6	3.5	3.2	100.0
The child’s father’s love for his partner is also evident in his efforts to enable and facilitate the best care for the child, including the child’s breastfeeding.	7.5	4.0	9.6	13.9	65.0	100.0
When a woman decides to breastfeed, she chooses between career and motherhood.	70.1	15.8	8.3	2.4	3.5	100.0

**Table 3:** Overview of the respondents’ answers to the knowledge scale BBAKQ prof questionnaire (N = 374).

The Knowledge Scale BBAKQ Prof Questionnaire	Percentage of Responses		
	True	False	Total
If the mother and child want to there is no reason for the mother not to breastfeed for two years or more.	90.9	9.1	100.0
The process of breastfeeding and the composition of breast milk are continuously adjusted to the needs of the child.	91.7	8.3	100.0
A mother with a fever may take some antipyretics (e.g., paracetamol) and breastfeed normally.	83.4	16.6	100.0
When the child vomits, it is necessary to stop breastfeeding.	20.3	79.7	100.0
By the time the baby has diarrhea, the mother can continue breastfeeding normally.	92.0	8.0	100.0
Many medications taken by the mother are not contraindicated during breastfeeding.	57.5	42.5	100.0
Breastfeeding mother is not allowed to drink one small cup of black coffee.	15.8	84.2	100.0
During the period when the breastfeeding mother is allowed to drink as much alcohol as she wants and it will not harm the health of the child.	4.8	95.2	100.0
At a time when the child has a bad cold and a high temperature, he does not need breast milk.	5.9	94.1	100.0
Child can be pampered by frequent breastfeeding.	11.8	88.2	100.0
Infant colic is more common in breastfed children.	12.6	87.4	100.0
The mother must stop breastfeeding immediately when she finds out she is pregnant.	9.4	90.6	100.0
While breastfeeding the mother is not allowed to go to the dentist.	3.7	96.3	100.0
When a breastfed child goes to hospital, the mother does not have to be with him.	5.9	94.1	100.0
Night breastfeeding causes tooth caries in a child.	6.2	93.9	100.0
Breastfeeding is a safe method of contraception.	5.1	94.9	100.0
Rooming in is important for establishing breastfeeding.	83.7	16.3	100.0
It is necessary to stop breastfeeding before going to the nursery or kindergarten.	18.5	81.6	100.0
UNICEF’s recommendation is that if it is appropriate for the mother and child to continue breastfeeding, there is no reason for mother not to breastfeed for two years or more.	90.9	9.1	100.0
The International Code of Marketing of Breast-milk Substitutes prohibits the advertising of breast milk substitutes in public.	44.9	55.1	100.0

**Table 4:** Overview of the results of the Mann–Whitney U Test for the results of respondents on the scales of behavior, attitudes, and knowledge and the overall respondents’ results in relation to occupation (nurse/other occupations) (N = 374).

Result on BBAKQ Prof. Questionnaire	Group	N	Mean Rank	Sum of Ranks	Mann–Whitney U	*p*-Value
Behavior scale	The nurses	334	189.6	63,276.0	6030.0	0.3
	The other occupations	40	171.2	6849.0		
Attitudes scale	The nurses	334	186.9	62,418.5	6473.5	0.8
	The other occupations	40	192.7	7706.5		
Knowledge scale	The nurses	334	188.1	62,813.5	6491.5	0.8
	The other occupations	40	182.8	7311.5		
Total	The nurses	334	188.8	63,042.0	6263.0	0.5
	The other occupations	40	177.1	7083.0		

## Supplementary Materials

The Breastfeeding behavior attitudes and knowledge questionnaire for health care staff is available online at https://vinkocatipovic.wixsite.com/my-site (accessed on 1 August 2022).
